# Fabrication of Superhydrophobic Coating Based on Waterborne Silicone-Modified Polyurethane Dispersion and Silica Nanoparticles

**DOI:** 10.3390/polym15010022

**Published:** 2022-12-21

**Authors:** Haidong Liu, Hengsen Xiong, Yongming Chang, Jianhui Xu, Chuanlai Xu, Yaolu Liu

**Affiliations:** 1 Key Laboratory of Testing Technology for Manufacturing Process, Ministry of Education, Southwest University of Science and Technology, Mianyang 621010, China; 2 Chengdu Kaimite Co., Ltd., No. 39 Jiancai Road, Chengdu 610051, China; 3 Chongqing Zhixiang Paving Technology Engineering Co., Ltd., China Merchants Chongqing Communications Technology Research and Design Institute, Chongqing 401336, China; 4 Sichuan Jiuzhou Electric Group Co., Ltd., No. 6 Jiuhua Road, Mianyang 621000, China; 5 Sichuan Avionics System Product Lightweight Design and Manufacturing Engineering Laboratory, Mianyang 621000, China; 6 Department of Engineering Mechanics, Chongqing University, Chongqing 400044, China

**Keywords:** superhydrophobic coating, eco-friendly, nano-composites

## Abstract

In this work, eco-friendly superhydrophobic coatings were prepared by dispersing hydrophobic silica nanoparticles and a waterborne silicone-modified polyurethane dispersion into an ethanol solution, which was free of fluorine and volatile toxic solvents. The effects of the silica content on the hydrophobicity and scratch resistance of the hydrophobic surfaces were investigated by WCA measurements and a sandpaper abrasion test, respectively. The experimental results indicated that when the silica content exceeded 30% by mass, the silica/silicone-modified polyurethane coatings had superhydrophobicity. Meanwhile, the superhydrophobic coatings with a silica content of 30% by mass simultaneously had the optimal mechanical stability. We studied the morphology and roughness of the hydrophobic surfaces with different silica content and attempted to briefly explain the influence mechanism of silica content. Furthermore, anti-icing and oil–water separation experiments were carried out on the superhydrophobic coatings, which exhibited good anti-icing performance and high separation efficiency. The eco-friendly superhydrophobic coating is expected to be applied in the fields of oil–water separation, anti-icing, and self-cleaning, etc.

## 1. Introduction

Superhydrophobic surfaces can prevent the adhesion of water droplets and provide the quick rolling-off of water droplets from the surface. Due to these abilities, superhydrophobic surfaces show enormous potential in many applications, such as anti-icing [1,2], anti-adhesion [3,4], oil–water separation [5,6], and self-cleaning [7,8,9] etc. Consequently, researchers have paid much attention to the development of construction methods for superhydrophobic surfaces [10,11,12].

Superhydrophobicity is achieved mainly in two ways, i.e., constructing micro/nanostructures and reducing the surface energy [13,14]. Some scholars focus on creating micro/nanostructures on hard materials—for example, metal [15], silicon [16], and Teflon [17]—through chemical etching, laser treatment, and hard-template methods, etc., followed by treatment with low-surface-energy compounds. These strategies can indeed produce robust superhydrophobic surfaces, which can exhibit long-term durability, while their disadvantages are also obvious, e.g., expensive equipment, time-consuming process, small treatment area, and limited substrate types [18]. These disadvantages limit the application of superhydrophobic surfaces with microstructures. Alternatively, superhydrophobic coatings break the constraints of material substrates and enable large-scale application; they are usually composed of a resin matrix and nanoparticles with low surface energy. A resin matrix, involving epoxy resins, acrylate resins, polyurethane, etc., is used to improve the mechanical properties and enhance the interfacial adhesion of superhydrophobic coatings. Nanoparticles such as silica (SiO_2_), zinc oxide (ZnO), carbon nanofibers (CNF), titanium dioxide (TiO_2_), carbon nanotube (CNT), aluminum oxide (Al_2_O_3_), and molybdenum disulfide (MoS_2_) are used to construct a roughened surface [19]. In most cases, to embed hydrophobic nanoparticles, it is necessary to dissolve the resin matrix as a dispersion, which usually requires a specific organic solvent, e.g., ethyl acetate [20], toluene/acetone [21]. The volatile nature of organic solvents is critical, as this provides rapid production and also contributes to the migration of nanoparticles to construct microstructures. However, some volatile organic solvents are toxic, or they may be non-toxic but emit unpleasant odors, which is not conducive to environmental protection and human health.

Water-based or waterborne coatings seem to be very promising for the construction of superhydrophobic coating layers in an environment friendly manner [19]. However, it is rather paradoxical to construct a water-repellent layer using an aqueous coating formula, since the water resistance of common waterborne or water-based resins with hydrophilic ionic groups in the main part is lower than those of solvent-based counterparts [22,23]. To increase the hydrophobicity of coatings composed of a water-based or waterborne resin, low-surface-energy materials, e.g., silicide and fluoride, can be incorporated into the material by grafting, spraying, or mixing [19]. For instance, Jing Zhang et al. successfully fabricated a superhydrophobic coating using a waterborne epoxy resin emulsion, while the superhydrophobic coating had less durable superhydrophobic properties due to the presence of hydrophilic groups. Meanwhile, the durable superhydrophobic properties of the coating could be improved by decreasing the number of hydrophilic groups in the coating by adding 1H, 1H, 2H, 2H-perfluorodecyltriethoxysilane (FAS-17), and 3-aminopropyltriethoxysilane (KH-550) to the formula [18]. Hao Zheng et al. prepared a durable and superhydrophobic waterborne polyurethane coating by spraying a mixed solution of waterborne polyurethane and fluoride-modified nano-SiO_2_ [24]. Zhao et al. dispersed silica nanoparticles directly in water merely with the assistance of low-surface-energy silanes. The resulted dispersion was used to treat fabric and produced superhydrophobic fabrics that can endure several cycles of washing [25]. Zhou et al. prepared a water-based, robust, self-healing superamphiphobic surface by stabilizing Teflon nanoparticles and fluorinated alkyl silane (FAS) in water with a commercial fluorocarbon surfactant. Due to the lubricating properties of Teflon nanoparticles and all fluorine-containing compounds, the resulting superhydrophobic coatings could retain an excellent superhydrophobic property against sand abrasion and scratching [26]. Zhao et al. prepared a superhydrophobic film by spraying waterborne fluorine-modified polyurethane. Fluorine was introduced into the waterborne polyurethane, which imparted low surface energy to the waterborne polyurethane films [27]. Chen and co-workers developed an all-water-based self-repairing superhydrophobic coating by incorporating FAS-12-loaded UV-responsive microcapsules and FAS-17-modified silica nanoparticles together into a polysiloxane latex. Upon UV light irradiation, the microcapsules could release FAS-12 as a low-surface-energy compound to restore the superhydrophobic properties of the coating damaged by physical abrasion [28]. A water-based fluorine-free superhydrophobic coating was reported by Ye et al., in which the silane groups were incorporated into acrylate copolymer chains through free radical polymerization and the ionized copolymers were further allowed to hydrolyze to yield silica nanoparticles, affording rough structures. This type of superhydrophobic coating showed good durability under harsh weather conditions [29]. Li et al. produced robust superhydrophobic coatings by spray-coating a polyurethane aqueous solution and a hexadecyl polysiloxane-modified SiO_2_ aqueous suspension onto substrates, using polyurethane resin as the adhesive. The coatings exhibited good static and dynamic anti-icing performance in outdoor environments [30]. Similarly, Zhao et al. reported a simple approach to the fabrication of waterborne, fluorine-free, and mechanically and thermally stable superhydrophobic coatings by spraying coatings composed of a waterborne suspension of hexadecyl polysiloxane-modified SiO_2_ and a waterborne PU solution [31].

Therefore, the preparation of superhydrophobic coatings with high durability can be considered by introducing fluoride and silicide in the aqueous coating formula. However, fluorocarbon compounds may destroy the protective ozone layer, which shields the Earth from harmful ultraviolet rays from the sun. Moreover, it has been shown that fluorocarbon compounds are toxic to human health [19]. On the other hand, polysiloxane, which has a number of interesting properties, such as low surface energy, good biocompatibility, and high thermal and oxidative stability, has been widely used to modify polyurethane materials [19]. A great deal of research on the improved thermal stability, tensile strength, solvent resistance, and water resistance obtained by introducing polysiloxane into waterborne polyurethane has been reported. However, very few studies focus on waterborne silicone-modified polyurethane dispersions to prepare superhydrophobic coatings.

In the present study, we successfully fabricated both fluorine-free and solvent-free superhydrophobic coatings using a waterborne silicone-modified polyurethane dispersion as an adhesive, which has a better hydrophobic property compared with common waterborne polymer dispersions such as polyurethane, epoxy resins, and acrylate resins. The water contact angle of coatings prepared from the pure waterborne silicone-modified polyurethane dispersion is 92.3 ± 1.8°, while the water contact angle of coatings prepared by common waterborne polymer dispersions is usually less than 90°. Meanwhile, the surface energy of the silicone-modified polyurethane dispersion is less than that of the most common waterborne polymer dispersions, which is beneficial for the preparation of superhydrophobic surfaces. Hydrophobic silica nanoparticles were employed to improve the hydrophobicity of the coatings by constructing micro- and nano-scale structures. Using the obtained silica/silicone-modified polyurethane suspension, hydrophobic surfaces with aluminum sheets, aluminum airfoil, and stainless-steel meshes as substrates were prepared by a spraying method.

We investigated the effect of silica content on the hydrophobicity of our hydrophobic surfaces through water contact angle (WCA) measurement. Micro- and nanoscale structural topography was obtained and analyzed using a scanning electron microscope (SEM) and atomic force microscope (AFM) for hydrophobic surfaces with different silica content. Sandpaper scratch tests were conducted to characterize the mechanical stability of hydrophobic surfaces with different silica content. Meanwhile, anti-icing and oil–water separation experiments were carried out on the aluminum airfoils and stainless-steel meshes with superhydrophobic coatings with a silica content of 30% by mass. The silica content of the superhydrophobic coating, with the optimal comprehensive performance, was obtained and might contribute to the application and promotion of eco-friendly superhydrophobic coatings.

## 2. Experimental Section

### 2.1. Materials

The waterborne silicone-modified polyurethane dispersion (RF-608) with enhanced hydrophobicity was a cosolvent-free aqueous polyurethane dispersion modified with silicone. The waterborne silicone-modified polyurethane dispersion was composed of carbinol silicone fluid (*C_OH_* = 56 mg KOH g^−1^), polyether glycol (*C_OH_* = 56 mg KOH g^−1^), 2,4-toluene diisocyanate (TDI), and other chemical agents. The waterborne silicone-modified polyurethane dispersion was purchased from Raffkni Import & Export Co., Ltd., Ningbo, China. Hydrophobic silica nanoparticles with size 30–70 nm was supplied by China Blue Star Chen Grand Chemical Co., Ltd, Chengdu, China. Ethanol was analytically pure and purchased from Tianjin Damao Chemical Reagent Co., Ltd., Tianjin, China.

### 2.2. Preparation of Superhydrophobic Coatings

The superhydrophobic coatings were fabricated through a simple and rapid spraying method. Briefly, silica nanoparticles and RF-608 were mixed in ethanol, with the five ratios shown in Table 1. In order to homogeneously disperse the two in ethanol, the mixture was placed into an ultrasonic washer at 29 kHz and 150 W for 30 min. Then, the mixture was sprayed on aluminum sheets, stainless-steel meshes, and airfoils. Finally, the hydrophobic coatings were obtained by curing the aluminum sheets, stainless-steel meshes, and airfoils in an oven at 60 °C for 30 min.

### 2.3. Characterization

The water contact angles (WCA) were measured with 3 μL drops of distilled water at room temperature with a DSA100 optical contact angle measuring instrument (Germany Cruise Co., Ltd., Hamburg, Germany). The value of the contact angle of the sample is reported as the average of measurements performed on three different spots on each sample. All measurements were taken three times and were reproducible to less than ±3°. The water sliding angle (WSA) was tested with 10 μL deionized water. The surface morphology of the hydrophobic coatings was characterized by an atomic force microscope (AFM) instrument (SPI4000 Probe Station, SIINT Instruments Co., Tokyo, Japan) in ambient conditions. The multimode AFM instrument was operated in a tapping mode. Olympus tapping-mode cantilevers with spring constants ranging from 51.2 to 87.8 N/m and the radius of the tip ranging from 6 to 10 nm (as specified by the manufacturer) were used. The roughness average was utilized to confirm the physical structures of the hydrophobic surfaces. The surface morphology was also investigated via scanning electron microscopy (SEM) with a JSM-5900LV instrument (FEI PHILIPS, Hillsboro, OR, USA) operated under an acceleration voltage of 5 kV for different samples. A thin layer of Au was sputter-coated on the samples in order to increase the conductivity.

The FTIR characterization was performed at ambient temperature with an infrared spectrometer (Thermo Scientific Nicolet 6700, Waltham, MA, USA).

**Figure 1:**
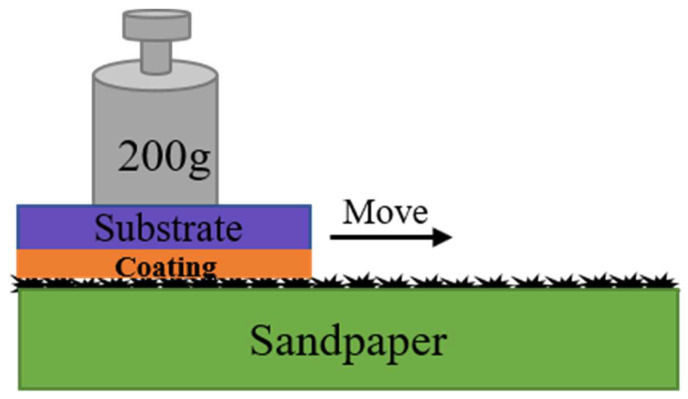
A schematic diagram of the sandpaper abrasion test. The green rectangle denotes the sandpaper, where the black protrusions illustrate that the upper part is its rough side. The purple rectangle represents the substrate, the aluminum sheet, and the orange below refers to the superhydrophobic coating sprayed on the aluminum sheet. The superhydrophobic coating was moved in a single direction under loading of 200 g.

The sandpaper abrasion test is a typical and efficient method for evaluating the robustness of superhydrophobic surfaces [32,33], and it was adopted to investigate the mechanical stability of the aluminum sheets with different silica content in our work, as shown in Figure 1. The superhydrophobic surface, with 1000-grit SiC sandpaper used as an abrasion surface, was moved at a constant speed of 5 mm/s in a single direction under a loading of 200 g. Movement of 10 cm was defined as one test cycle. WCAs were measured before and after each test cycle, and the mechanical stability of the coatings was evaluated according to WCA changes.

**Figure 2:**
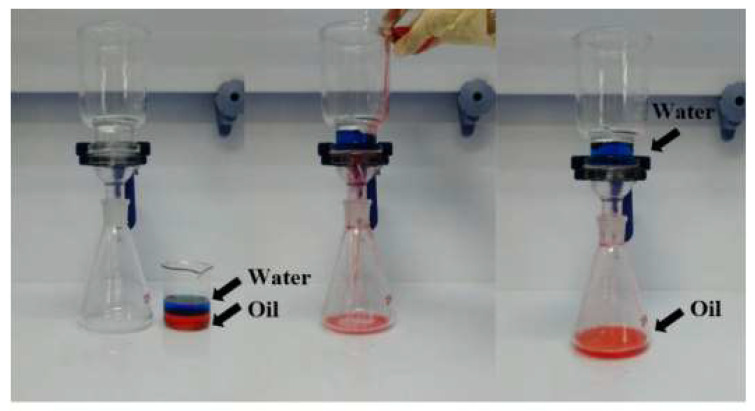
Photos of the oil–water separation experiment. The left, middle, and right are before, during, and after separation, respectively. The oil–water mixture, where the blue liquid is water and the red is oil, was poured onto the superhydrophobic stainless-steel mesh. It was observed that water was trapped on the stainless-steel mesh and oil flowed down through the stainless-steel mesh.

The oil–water separation experiment was carried out using 100-mesh stainless-steel meshes, which were sprayed with superhydrophobic coatings containing 30% silica content. The oil–water mixture was prepared according to the method described in the literature [26]. The experimental set-up and process for oil–water separation are shown in Figure 2, where oil and water were dyed red and blue, respectively, to achieve the best observations. The oil–water mixture was poured onto the superhydrophobic sample to test its oil–water separation performance, and this process was cycled on the same sample.

For icing wind tunnel tests, small-scale airfoils with a superhydrophobic coating were tested inside a low-speed small-scale closed-loop icing wind tunnel [34,35]. The experiment was performed at a true air speed of 30 m s^−1^, a static air temperature of −5 °C, and liquid water content of 0.2 g m^−3^.

## 3. Results and Discussion

### 3.1. Surface Wettability of Coatings

**Figure 3:**
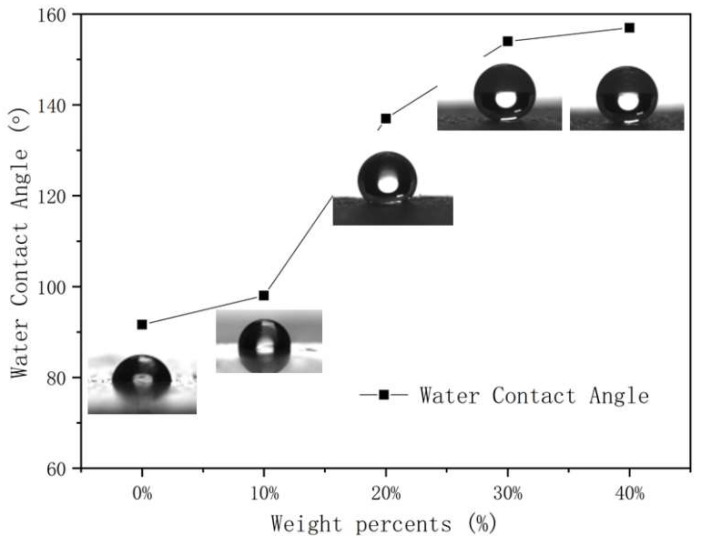
Water contact angle with respect to silica content by mass is illustrated for the aluminum sheets with hydrophobic coatings. The WCA increases with silica content, and photos of water droplets on hydrophobic surfaces with different silica content are shown accordingly.

**Figure 4:**
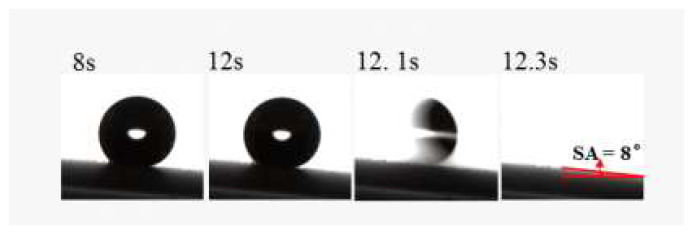
Measurement of water sliding angle for superhydrophobic coatings with 30% silica content is shown, in which the WSA is obtained as 8°. Specifically, when the inclination angle of the superhydrophobic surface is 8°, the water droplets easily roll off the surface.

The surface wettability was investigated in the superhydrophobic coatings with different silica content by measuring the WCA and WSA. Figure 3 depicts the WCA variation with the silica content in the coatings, and the effect of silica content on the SiO_2_/RF-608 composite. The WCA of pure RF-608 is 92.3 ± 1.8°, which implies that the waterborne silicone-modified polyurethane adopted has a certain intrinsic hydrophobicity. Surfaces with a WCA higher than 150° and WSA lower than 10° are defined as superhydrophobic surfaces. When the silica content is equal to or more than 30%, the WCA of the coating exceeds 150°. This indicates that the coating can be transformed from a hydrophobic surface to a superhydrophobic surface with increasing silica content. In particular, the WCA of the coatings reaches 157.8 ± 2.1° when the silica content is around 40%. In addition, Figure 4 illustrates the WSA measurement for superhydrophobic coatings with 30% silica content and its WSA = 8°, which satisfies the definition of superhydrophobicity.

### 3.2. Surface Morphology of Coatings with Different Silica Content

**Figure 5:**
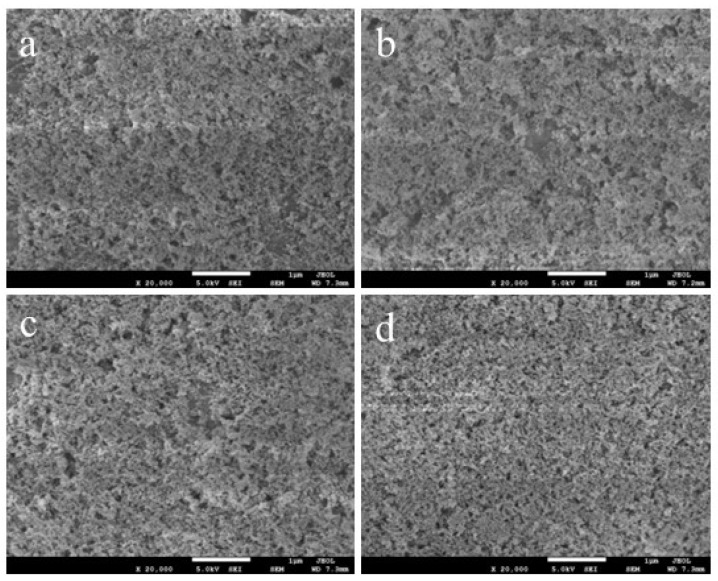
Microstructure images of hydrophobic coatings with silica content of 10%, 20%, 30%, and 40% are presented in (**a**–**d**).

**Figure 6:**
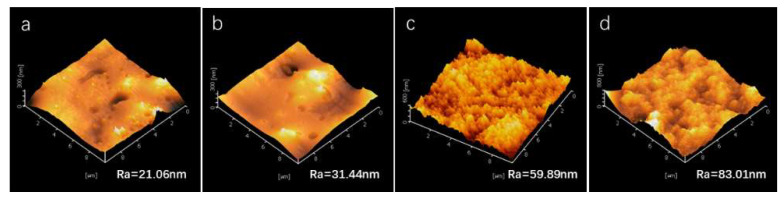
Roughness measurements of hydrophobic coatings with silica content of 10%, 20%, 30%, and 40% are illustrated in (**a**–**d**). These measurements were obtained by AFM, and the average mean square roughness values (Ra) of coatings with silica content of 10%, 20%, 30%, and 40% are 21.6 nm, 31.44 nm, 59.89 nm, and 83.01 nm, respectively.

Micro- and nano-scale structures make significant contributions to the preparation of superhydrophobic surfaces. SEM images of the microstructures of SiO_2_/RF-608 coatings with different silica content are presented in Figure 5. Many random, isolated, micro-scale aggregates of silica are observed in the surfaces with 10% and 20% silica content, as shown in Figure 5a,b, respectively, and these microstructures contribute to the improvement of the hydrophobic properties. In addition, the number and size of aggregates increase with the silica content, which can be seen in Figure 5c,d. Meanwhile, the aggregates gradually develop into a continuous phase as the silica content increases from 30% to 40%. Combined with Figure 3, when the silica content reaches 30%, the coating starts to exhibit superhydrophobicity. This may be attributed to the fact that networks of silica aggregates are developed in coatings with high silica content (>30%). Moreover, the superhydrophobicity is greatly enhanced when further constructing rich networks with increasing silica content. To further investigate the micro- and nano-scale roughness of our hydrophobic surfaces with different silica content, we used AFM to obtain images of the surface topography and measure the average mean square roughness (Ra), which are presented in Figure 6. The Ra values of hydrophobic surfaces with silica content of 10%, 20%, 30%, and 40% are 21.6 nm, 31.44 nm, 59.89 nm, and 83.01 nm, respectively. The Ra increases with the silica content, which is beneficial for the enhancement of hydrophobicity.

### 3.3. FTIR Analysis

**Figure 7:**
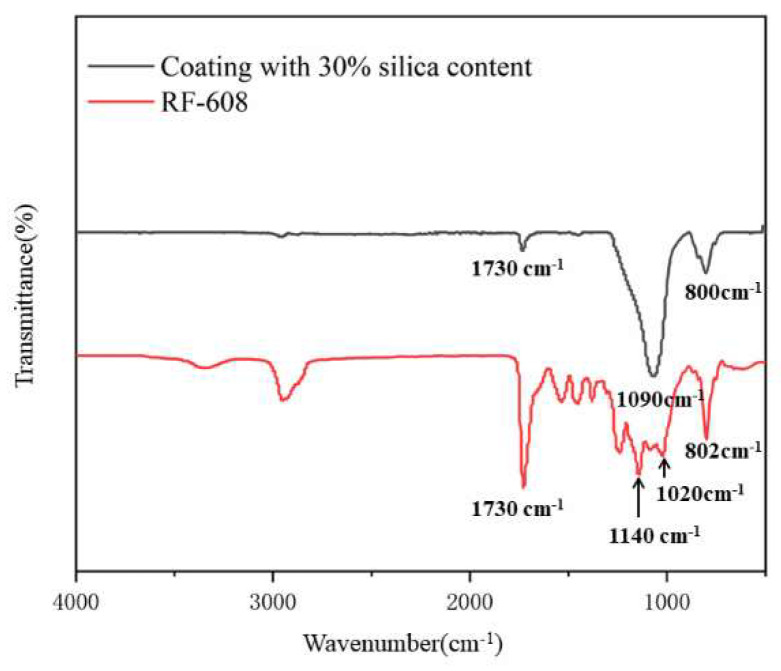
FTIR spectra of coatings with 30% silica content and pure RF-608.

Figure 7 shows the FTIR-IR spectra of the pure RF-608 and coating with silica content of 30%. It can be seen that characteristic absorption peaks of –Si–CH3, –Si–O–Si–, C–O–C, and –NHCO– appeared at 802 cm^−1^, 1020 cm^−1^, 1140 cm^−1^, and 1730 cm^−1^, which indicates that the carbinol silicone fluid accessed the polymer chain successfully. Meanwhile, the peaks of 800 cm^−1^ and 1090 cm^−1^ present the characteristic absorption of Si–O–Si, which means that SiO_2_ was well dispersed in the matrix of the waterborne silicone-modified polyurethane [36,37].

### 3.4. Sandpaper Abrasion Test

Table 2 shows the WCA for coatings with different silica content before and after abrasion. The scratch resistance of hydrophobic coatings decreases with increasing silica content. Therefore, when the coating achieves the superhydrophobic condition, the silica content should be reduced as much as possible to ensure sufficient scratch resistance.

### 3.5. Oil–Water Separation

Our eco-friendly superhydrophobic coatings are not only non-toxic; they are also both water-repellent and lipophilic. These properties provide them with the potential to yield unusually high results in oily effluent separation. As shown in Figure 2, when the oil–water mixture was poured on the superhydrophobic coated mesh, the red oil droplets continuously dropped into the conical flask through the mesh, while the blue water was trapped in the funnel by the superhydrophobic coated mesh because of its water repellence. The oil–water separation experiment was cyclically conducted on the same superhydrophobic coated mesh, which finally exhibited separation efficiency of over 92%, indicating good reproducibility for the oil–water separation effect of superhydrophobic coated meshes.

### 3.6. Anti-Icing Performance

**Figure 8:**
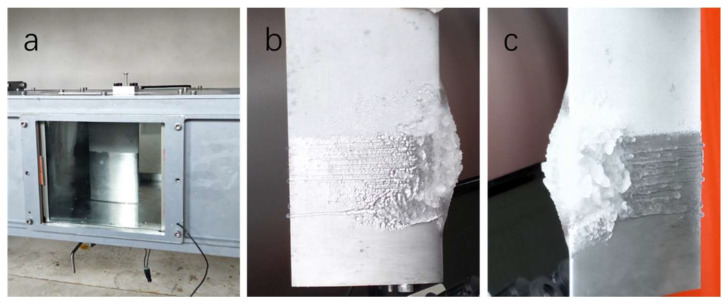
Top view before the experiments (**a**). Ice accretion on an airfoil with different angles (**b**,**c**).

Figure 8 highlights the results of the ice accretion on the airfoil before icing and 250 s after the icing cloud was introduced. For the experiments performed at TAS = 30 m s^−1^ and SAT = −5 °C, the ice accretion on the top half of an airfoil coated with the superhydrophobic coating with 30% silica content was significantly reduced compared to the dense ice layer formed on the bottom half of the airfoil as shown Figure 8b,c. Moreover, no ice formation was detected on the top half of the airfoil with the superhydrophobic coating for up to 25 s after activating the ice cloud.

## 4. Conclusions

In this work, eco-friendly superhydrophobic coatings were fabricated using an inherently hydrophobic waterborne silicone-modified polyurethane dispersion, RF-608, which has significant advantages of being fluorine-free and solvent-free. Sandpaper abrasion tests and oil–water separation and anti-icing experiments were performed on superhydrophobic coatings with different silica content. We found that the superhydrophobic coating with silica content of 30% by mass exhibits excellent scratch resistance, efficient oil–water separation performance, and passive anti-icing performance, and thus may have broad application prospects in the fields of anti-icing, oil–water separation, self-cleaning, etc.

## Figures and Tables

**Table 1:** SiO_2_/waterborne polyurethane hydrophobic coating formula.

Ingredient	Phr ^a^
RF-608	100
SiO_2_	0, 10, 20, 30, 40
Ethanol	100

^a^ represents parts per hundred of RF-608 by mass.

**Table 2:** WCA of SiO_2_/RF-608 composite before and after abrasion.

Silica Content	10%	20%	30%	40%
WCA before abrasion	98.4° ± 1.8	137.3° ± 1.6	154.4° ± 2.6	157.8° ± 2.1
WCA after abrasion	97.6° ± 1.8	135.3° ± 1.9	151.3° ± 1.1	147.4° ± 2.8

## Data Availability

Not applicable.

## References

[1] Zheng W.W., Teng L., Lai Y.K., Zhu T.X., Li S.H., Wu X.W., Cai W.L., Chen Z., Huang J.Y. (2022). Magnetic responsive and flexible composite superhydrophobic photothermal film for passive anti-icing/active deicing. Chem. Eng. J..

[2] Li W., Zhan Y.L., Yu S.R. (2021). Applications of superhydrophobic coatings in anti-icing: Theory, mechanisms, impact factors, challenges and perspectives. Prog. Org. Coat..

[3] Foorginezhad S., Zerafat M.M. (2019). Fabrication of stable fluorine-free superhydrophobic fabrics for anti-adhesion and self-cleaning properties. Appl. Surf. Sci..

[4] Mao Y., Wang F., Li Y., Guidoin R., Wang L., Wang F.J. (2020). Facile fabrication of potent superhydrophobic surface on physical barriers with enhanced anti-adhesion efficiency. Appl. Surf. Sci..

[5] Ren Z.Y., Wen G., Guo Z.G. (2019). Biomimetic high-intensity superhydrophobic metal rubber with anti-corrosion property for industrial oil-water separation. New J. Chem..

[6] Deng W., Fan T.Z., Li Y. (2021). In situ biomineralization-constructed superhydrophilic and underwater superoleophobic PVDF-TiO_2_ membranes for superior antifouling separation of oil-in-water emulsions. J. Membr. Sci..

[7] Yoon H., Kim H., Latthe S.S., Kim M.W., Al-Deyab S., Yoon S.S. (2015). A highly transparent self-cleaning superhydrophobic surface by organosilane-coated alumina particles deposited via electrospraying. J. Mater. Chem. A.

[8] Toma M., Loget G., Corn R.M. (2014). Flexible Teflon Nanocone Array Surfaces with Tunable Superhydrophobicity for Self-Cleaning and Aqueous Droplet Patterning. ACS Appl. Mater. Inter..

[9] Zhang X.T., Liu S.Q., Salim A., Seeger S. (2019). Hierarchical Structured Multifunctional Self-Cleaning Material with Durable Superhydrophobicity and Photocatalytic Functionalities. Small.

[10] Xue C.H., Jia S.T., Zhang J., Ma J.Z. (2010). Large-area fabrication of superhydrophobic surfaces for practical applications: An overview. Sci. Technol. Adv. Mater..

[11] Qu M.N., Zhang B.W., Song S.Y., Chen L., Zhang J.Y., Cao X.P. (2007). Fabrication of superbydrophobic surfaces on engineering materials by a solution-immersion process. Adv. Funct. Mater..

[12] Kim M.K., Yao W.H., Cho Y.R. (2022). Fabrication of superhydrophobic surface with hierarchical structure by thermal imprinting and spraying. Colloid Surf. A.

[13] Yan Y.Y., Gao N., Barthlott W. (2011). Mimicking natural superhydrophobic surfaces and grasping the wetting process: A review on recent progress in preparing superhydrophobic surfaces. Adv. Colloid Interfac..

[14] Zhang Y.L., Xia H., Kim E., Sun H.B. (2012). Recent developments in superhydrophobic surfaces with unique structural and functional properties. Soft Matter..

[15] Emelyanenko A.M., Shagieva F.M., Domantovsky A.G., Boinovich L.B. (2015). Nanosecond laser micro- and nanotexturing for the design of a superhydrophobic coating robust against long-term contact with water, cavitation, and abrasion. Appl. Surf. Sci..

[16] Hoshian S., Jokinen V., Somerkivi V., Lokanathan A.R., Franssila S. (2015). Robust Superhydrophobic Silicon without a Low Surface-Energy Hydrophobic Coating. ACS Appl. Mater. Interfaces.

[17] Scarratt L.R.J., Hoatson B.S., Wood E.S., Hawkett B.S., Neto C. (2016). Durable Superhydrophobic Surfaces via Spontaneous Wrinkling of Teflon AF. ACS Appl. Mater. Interfaces.

[18] Li M., Li Y., Xue F., Jing X.L. (2018). Water-based acrylate copolymer/silica hybrids for facile preparation of robust and durable superhydrophobic coatings. Appl. Surf. Sci..

[19] Zhao H., Gao W.C., Li Q., Khan M.R., Hu G.H., Liu Y., Wu W., Huang C.X., Li R.K.Y. (2022). Recent advances in superhydrophobic polyurethane: Preparations and applications. Adv. Colloid Interfaces.

[20] Zhang Z.Z., Ge B., Men X.H., Li Y. (2016). Mechanically durable, superhydrophobic coatings prepared by dual-layer method for anti-corrosion and self-cleaning. Colloid Surface A.

[21] Atta A.M., Al-Lohedan H.A., Ezzat A.O., Al-Hussain S.A. (2016). Characterization of superhydrophobic epoxy coatings embedded by modified calcium carbonate nanoparticles. Prog. Org. Coat..

[22] Ge Z., Luo Y.J. (2013). Synthesis and characterization of siloxane-modified two-component waterborne polyurethane. Prog. Org. Coat..

[23] Liu D., Wu G.M., Kong Z.W. (2017). Preparation and characterization of a polydimethylsiloxane-modified, epoxy-resin-based polyol dispersion and its crosslinked films. J. Appl. Polym. Sci..

[24] Zheng H., Pan M.W., Wen J., Yuan J.F., Zhu L., Yu H.F. (2019). Robust, Transparent, and Superhydrophobic Coating Fabricated with Waterborne Polyurethane and Inorganic Nanoparticle Composites. Ind. Eng. Chem. Res..

[25] Zhao Q., Wu L.D.Y.L., Huang H., Liu Y.C. (2016). Ambient-curable superhydrophobic fabric coating prepared by water-based non-fluorinated formulation. Mater. Des..

[26] Zhou H., Wang H.X., Niu H.T., Zhao Y., Xu Z.G., Lin T. (2017). A Waterborne Coating System for Preparing Robust, Self-healing, Superamphiphobic Surfaces. Adv. Funct. Mater..

[27] Zhao B., Jia R.P. (2019). Preparation of super-hydrophobic films based on waterborne polyurethane and their hydrophobicity characteristics. Prog. Org. Coat..

[28] Chen K.L., Zhou S.X., Yang S., Wu L.M. (2015). Fabrication of All-Water-Based Self-Repairing Superhydrophobic Coatings Based on UV-Responsive Microcapsules. Adv. Funct. Mater..

[29] Ye H., Zhu L.Q., Li W.P., Liu H.C., Chen H.N. (2017). Simple spray deposition of a water-based superhydrophobic coating with high stability for flexible applications. J. Mater. Chem. A.

[30] Li Y.B., Li B.C., Zhao X., Tian N., Zhang J.P. (2018). Totally Waterborne, Nonfluorinated, Mechanically Robust, and Self-Healing Superhydrophobic Coatings for Actual Anti-Icing. ACS Appl. Mater. Interfaces.

[31] Zhao X., Li Y.B., Li B.C., Hu T., Yang Y.F., Li L.X., Zhang J.P. (2019). Environmentally benign and durable superhydrophobic coatings based on SiO2 nanoparticles and silanes. J. Colloid Interfaces Sci..

[32] Su F.H., Yao K. (2014). Facile Fabrication of Superhydrophobic Surface with Excellent Mechanical Abrasion and Corrosion Resistance on Copper Substrate by a Novel Method. ACS Appl. Mater. Interfaces.

[33] Lu Y., Sathasivam S., Song J.L., Crick C.R., Carmalt C.J., Parkin I.P. (2015). Robust self-cleaning surfaces that function when exposed to either air or oil. Science.

[34] De Pauw D., Dolatabadi A. (2017). Effect of Superhydrophobic Coating on the Anti-Icing and Deicing of an Airfoil. J. Aircr..

[35] Alamri S., Vercillo V., Aguilar-Morales A.I., Schell F., Wetterwald M., Lasagni A.F., Bonaccurso E., Kunze T. (2020). Self-Limited Ice Formation and Efficient De-Icing on Superhydrophobic Micro-Structured Airfoils through Direct Laser Interference Patterning. Adv. Mater. Interfaces.

[36] Du Y., Zhang J., Zhou C. (2016). Synthesis and properties of waterborne polyurethane-based PTMG and PDMS as soft segment. Polym. Bull..

[37] Wu Z., Wang H., Tian X., Cui P., Ding X., Ye X. (2014). The effects of polydimethylsiloxane on transparent and hydrophobic waterborne polyurethane coatings containing polydimethylsiloxane. Phys. Chem. Chem. Phys..

